# Dry Ageing Effect on Beef Quality Characteristics from Raramuri Criollo vs. Hereford × Angus

**DOI:** 10.3390/ani16111716

**Published:** 2026-06-04

**Authors:** Tlacaélel De la Cruz-Torres, Mariana Huerta-Jimenez, Alma D. Alarcón-Rojo, Felipe A. Rodríguez-Almeida, Iván A. García-Galicia

**Affiliations:** 1 Facultad de Zootecnia y Ecología, Universidad Autónoma de Chihuahua, Chihuahua 31000, Mexico; tlacam50@gmail.com (T.D.l.C.-T.); mhuertaj@uach.mx (M.H.-J.); frodrigu@uach.mx (F.A.R.-A.); 2 Centro de Enseñanza, Investigación y Extensión en Ganadería Tropical (C.E.I.E.G.T.), Facultad de Medicina Veterinaria y Zootecnia, Universidad Nacional Autónoma de México, Tlapacoyan 93650, Mexico

**Keywords:** indigenous breeds, sustainable meat, meat quality, sustainable livestock systems, arid rangelands

## Abstract

Raramuri Criollo (RC) cattle from Mexico have special genetic and resilience characteristics which have positioned them as an important alternative for future meat production under arid conditions. Despite the remarkable hardiness of RC cattle, their meat has been anecdotally catalogued as lean, tough and unattractive for consumption. However, abundant information about RC meat quality has not been documented to date. This study evaluated technological characteristics and tenderness of *Longissimus lumborum* muscle from Raramuri Criollo cattle compared to a commercial Hereford × Angus (H × A) crossbreed during dry ageing. pH, water-holding capacity (WHC), Warner–Bratzler shear force (SF), and instrumental colour were analyzed at 0, 15 and 30 d of ageing. pH values remained within the normal physiological range (5.4–5.5) for high-quality beef in both genetic groups. WHC showed significant differences among days of ageing, but not by racial group (*p* > 0.05). Additionally, RC beef demonstrated higher chromatic stability than H × A meat during ageing. Meat from H × A animals exhibited faster early tenderization, while RC beef showed a more gradual and sustained reduction in SF, with RC reaching lower SF than meat from crossbred animals by d 30 of ageing. From day 0 to day 15 SF decreased by 9.48 N and 12.16 N for RC and H × A meat, respectively, while from day 16 to day 30 the SF showed decreases of 4.73 and 2.11 N for RC and H × A meat respectively. In conclusion, RC meat is as tender as H × A after 30 d ageing without deterioration in its technological properties. These findings highlight the potential of RC to produce competitive tender meat, supporting valorisation of indigenous cattle genetic resources as a viable strategy for sustainable beef production in arid environments.

## Simple Summary

Raramuri Criollo (RC) cattle from Mexico have special genetic and resilience characteristics but very little information about their meat quality is documented to date. This study evaluated technological and quality characteristics of muscle from RC cattle compared to a commercial Hereford × Angus crossbreed during dry ageing. Ageing significantly improved quality parameters during the first 15 d. While crossbred animals exhibited faster early tenderization, RC beef showed a more gradual and sustained tenderization, reaching lower shear force than crossbred animals by d 30 of ageing. Additionally, RC demonstrated improved colour stability during storage. In conclusion, RC meat is tender and it retains its technological properties after ageing. Raramuri Criollo cattle farming is a viable strategy for sustainable beef production in arid environments.

## 1. Introduction

Beef quality is a multifactorial trait determined by complex interactions among genetic background, production systems, muscle biochemistry, and *postmortem* processing conditions [1]. Cattle breeds have intensively been selected for rapid growth, carcass yield, and early development of desirable meat quality traits. However, the emphasis on productivity has often marginalized locally adapted cattle populations that may possess valuable adaptive and productive traits relevant to emerging sustainability challenges [2].

Criollo cattle represent a group of indigenous bovine populations distributed throughout the Americas with centuries of natural and semi-natural selection under harsh environmental conditions, like arid and semi-arid ecosystems. As a result, they exhibit remarkable resilience, efficient utilization of marginal grazing resources, and high adaptability to environmental stressors [2,3]. Raramuri Criollo (RC) cattle have historically been overlooked by the commercial beef industry in the North of Mexico due to their small size, low weights, and non-uniform conformation. In terms of quality, their meat is anecdotally described as tough, not very juicy, and very lean, likely due to traditional and inadequate slaughter and meat preservation practices in their original communities. Nevertheless, RC have sparked research interest by their remarkable capacity for survival and adaptation to limited resources, being able to survive on woody plants, cacti, and native herbaceous vegetation largely limited in abundance due to low and erratic rainfall [2,4,5]. In recent years, the productive, carcass, and sensory characteristics of Criollo vs Brangus crossbreed meat have begun to be evaluated [6,7]. Brangus, a 5/8 Angus and 3/8 Brahman crossbreed with good carcass quality and resilient enough to endure the harsh conditions of arid environments [8]. However, RC meat characteristics are scarcely documented. Understanding the biochemical and technological behaviour of RC meat is essential for developing value-added production strategies able to improve their economic competitiveness while supporting the conservation of animal genetic resources.

Tenderness, colour stability, and water-holding capacity (WHC) are relevant meat attributes to consumer acceptance. They play a central role in shaping purchasing decisions and overall eating satisfaction [9,10]. Further, the three characteristics are strongly influenced by *postmortem* biochemical processes occurring during ageing, such as proteolysis and structural modifications of myofibrillar proteins [11,12]. Dry ageing is one of the most traditional methods for enhancing beef quality, involving the controlled storage of unpackaged meat under low temperature, regulated humidity, and continuous airflow. Which allows enzymatic proteolysis and moisture evaporation to concentrate flavour, intensify aroma, and increase tenderness [11,13]. Nevertheless, the response of beef to dry-ageing processes can vary substantially depending on genetic background, muscle structure, intramuscular fat content, and metabolic characteristics. Differences among breeds may influence the activity of endogenous proteolytic systems, oxidative stability, and water distribution within muscle tissue, affecting the rate and extent of tenderization and colour stability in *postmortem* storage [14,15]. Therefore, the objective of this study was to evaluate the meat quality attributes of the *Longissimus lumborum* muscle from RC cattle compared with a commercial Hereford × Angus breed before and after dry ageing for 30 d *postmortem*.

Specifically, this research analyzed changes in pH, WHC, Warner–Bratzler shear force, and instrumental colour during different ageing periods to better understand the *postmortem* quality dynamics of these contrasting genetic groups. By elucidating these mechanisms, this pilot study aims to contribute to the scientific understanding of meat quality in indigenous cattle and to support the sustainable valorisation of Criollo genetic resources in beef production systems.

## 2. Materials and Methods

### 2.1. The Animals and the Meat Samples

The animal study protocol was approved by the Postgraduate Department and by the Animal Ethics and Welfare Committee of the Faculty of Animal Science and Ecology of the Autonomous University of Chihuahua (UACH). This study was carried out using cattle from the Research and Technology Transfer Centre, UACH, located in Teseachi, Chihuahua, Mexico (28°48′ N; 107°25′ E). Sixteen steers (430–520 kg) were used in this exploratory study. Ten animals were Raramuri Criollo (RC) and six were the Hereford × Angus crossbreed (H × A). The animals were born and raised in the Research Centre, grazing native pastures including vegetable species such as *Bouteloua gracilis*, *Bouteloua hirsuta*, *Muhlenbergia rigida*, *Lycurus phleoides*, and *Elynorus barbiculmis*. After reaching 14 m old, they were allowed to graze for two h/d in meadows based on alfalfa and ryegrass until they reached 20 m old. Later, the animals were transported to the city of Chihuahua, Mexico, to be fattened for 3 months with a concentrated diet (70% grain and 30% corn straw, 18% crude protein) under indoor conditions, after a one-month adaptation period [16]. Animals were slaughtered (~24 m old) in the municipal slaughterhouse of Chihuahua city according to the Official Mexican Regulations [17]. After 48 h *postmortem* storage under refrigeration (4 °C), the whole *Longissimus lumborum* (*Llm*) muscle (n = 16; 32–36 cm length) was separated from each carcass and used for this study. Each muscle was divided into three portions (10–12 cm thick), and the excess of subcutaneous fat was manually removed prior to ageing. Every one-third portion of the muscles was randomly assigned to each ageing period (0, 15, or 30 d); hence, every muscle was aged over each of the three periods.

The ageing process consisted of placing the 48 portions (3 from each loin) of meat in a Dry Ager DX 500 meat ageing unit (Landig + Lava GmbH & Co. KG, Dry Ager Manufactur Mkstrab 90, Bad Saulgau, Germany). This unit was equipped with temperature control (1.5–2.0 °C), relative humidity control (75–80%), and ultraviolet light for the elimination of contaminating microorganisms. The exterior lighting of the ageing cabinet was LED with a light spectrum without ultraviolet radiation, generating minimal heat that did not affect the core temperature of the meat [18].

Samples were removed from the chamber after the corresponding ageing times for physicochemical analysis. All determinations were performed in triplicate except for texture, for which at least six measurements were taken on each sample (one-third portion of muscle).

### 2.2. pH

The pH of the beef was recorded at 45 min and 24 h *postmortem* directly on the carcass, as well as at days 0, 15 and 30 of ageing, using a digital pH meter (CR-400 Hanna, Cluj-Napoca, Romania), previously calibrated according to the manufacturer’s instructions. The measurements of aged meat were made directly on the beef steaks according to the method of Korkeala et al. [19].

### 2.3. Water-Holding Capacity (WHC)

The WHC was determined by the press method [20] as modified by Tsai & Ockerman [21]. A sample of approximately 0.5 g was weighed on an analytical balance and placed between two filter papers (Number 1, 15 mm pore diameter, Whatman, Sigma, Roedermark, Germany), which in turn were placed between two plexiglass plates (15 × 15 cm), upon which a constant 10 kg pressure was applied for 15 min. WHC was expressed as percent difference in sample weight before and after compression.

### 2.4. Warner–Bratzler Shear Force

Determination of the SF was carried out according to the AMSA methodology [22]. The steak samples were cooked on electric grills (George Foreman^®^, Middleton, WI, USA) until reaching 72 °C at the geometrical centre of the sample. Cooked samples were tempered at room temperature and left to cool at 4 °C overnight. After 24 h, cylinders 12.7 mm in diameter were cut parallel to the muscle fibres using an electric hole punch. Cored samples were sheared using a TA.XT-plus texture analyser (Stable Micro Systems, Godalming, Surrey, UK) with a V-shaped blade attached to a 100 N load cell at a crosshead speed of 200 mm/min. Shear force (SF) values, reported in Newtons, were determined from the average of six replicates for each sample.

### 2.5. Colour

Surface colour of all muscles was measured in triplicate at three positions on every steak with a Minolta Chromameter (Konica Minolta Camera, Basildon, UK. Aperture, 8 mm. Illuminant C, D65. Standard observer, C: Y = 94.2, x = 0.3130 and y = 0.3190). Following the CIE Lab methodology [23], L* (lightness), a* (redness), and b* (yellowness) coordinates were measured. The meter was calibrated using a Minolta calibration ceramic tile (L* = 93.8, a* = 0.3136, b* = 3192). Also, colour intensity/saturation (C*) was calculated (a*2 + b*2)^½^. The measurements were performed on parts of the sample that were free of visible connective and adipose tissue, allowing the meat to bloom for 45 min prior to analysis at 20 °C.

### 2.6. Statistical Analyses

Statistical analyses were conducted using a 2 × 3 (genetic group × ageing time) multifactorial analysis of variance (ANOVA) within a General Linear Model (GLM) procedure of SAS (Statistical Analysis System, 9.4) with the individual animal as the experimental unit. The statistical model tested main effects and all two-way interactions. When statistical differences were detected with ANOVA (*p* < 0.05), Tukey’s test was performed for mean comparisons. Differences were considered significant when *p* < 0.05.

## 3. Results

### 3.1. pH

**Figure 1:**
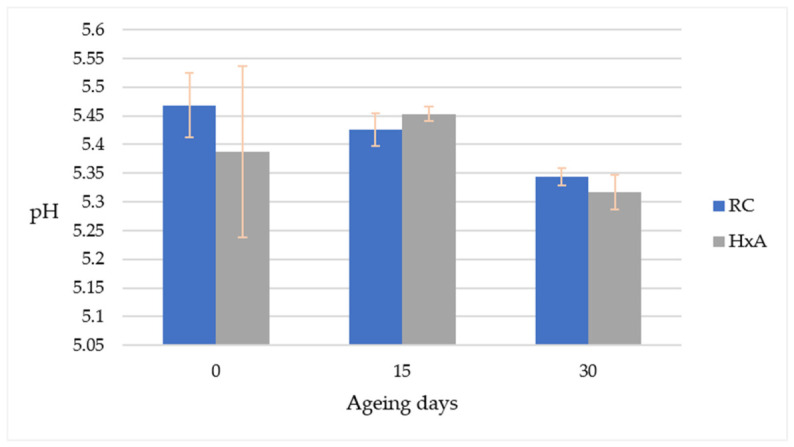
Changes in pH beef from Raramuri Criollo (RC) and Hereford × Angus crossbred (H × A) cattle during ageing (0, 15, and 30 d). The data are expressed as means ± standard errors.

No significant effects were detected for breed group (*p* = 0.567), ageing duration (*p* = 0.096), or the breed × ageing interaction (*p* = 0.637) on the pH of the *Llm* (Figure 1). Across treatments, mean pH values ranged from 5.4 to 5.8, consistent with values typically reported for high-quality beef and indicative of adequate glycogen reserves and appropriate pre-slaughter handling conditions [24]. Although Group H × A presented a slightly lower initial pH (5.38), values for both groups remained within the normal physiological range throughout the ageing period.

### 3.2. Water-Holding Capacity

**Figure 2:**
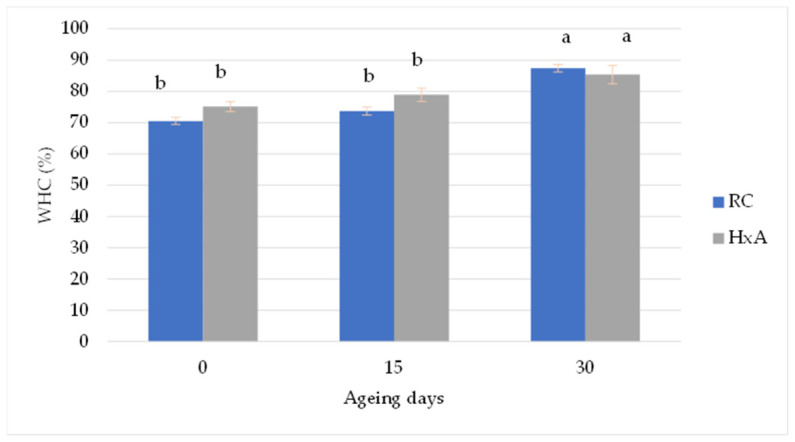
Changes in water-holding capacity (WHC) of beef from Raramuri Criollo (RC) and Hereford × Angus crossbred (H × A) cattle during ageing (0, 15 and 30 d). The data are expressed as means ± standard errors. Different letters (a, b) indicate significant differences among storage times within the same animal group (*p* < 0.05).

No significant differences were observed between genetic groups (*p* = 0.064) nor for the breed × ageing day interaction (*p* = 0.063) in WHC. In contrast, ageing duration exerted a significant effect (*p* < 0.001). WHC remained relatively stable between 0 and 15 d of ageing (*p* = 0.089), but a significant increase was observed between 15 and 30 d (*p* < 0.001; Figure 2).

### 3.3. Warner–Bratzler Shear Force

**Figure 3:**
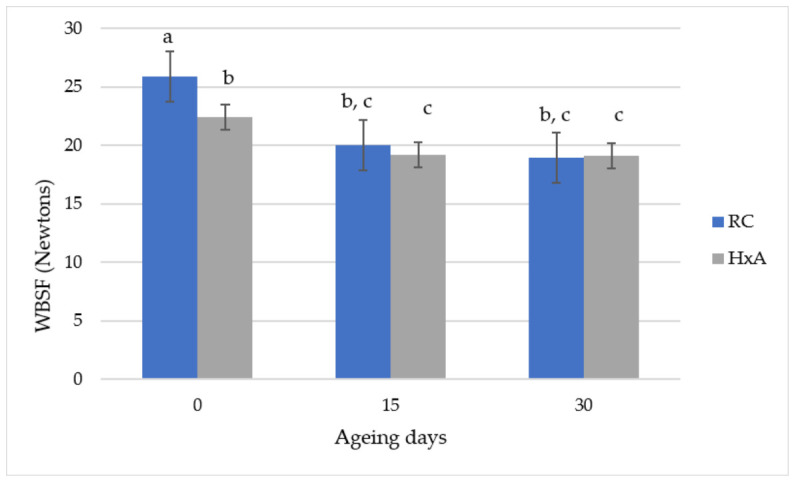
Warner–Bratzler shear force of beef from Raramuri Criollo (RC) and Hereford × Angus crossbred (H × A) cattle during ageing (0, 15, and 30 d). The data are expressed as means ± standard errors. Different letters (a, b, c) indicate significant differences among storage times within the same animal group (*p* < 0.05).

The SF of the cooked beef was significantly affected by the genetic group (*p* = 0.006) (Figure 3), ageing duration (*p* < 0.001), and the interaction of genetic group × day of ageing (*p* = 0.006). At 0 d of ageing, the beef from the European crossbreed exhibited slightly lower SF than beef from RC, indicating higher initial tenderness. By day 15, both groups showed a significant reduction in SF (*p* < 0.001), reaching similar tenderness levels.

Hereford × Angus (H × A) beef achieved its lowest SF values by day 15, remaining relatively stable thereafter until 30 d of ageing. In contrast, RC beef continued to exhibit a gradual decline in SF during the extended ageing period, catching up in tenderness to the crossbreed by day 30 (Figure 3).

**Figure 4:**
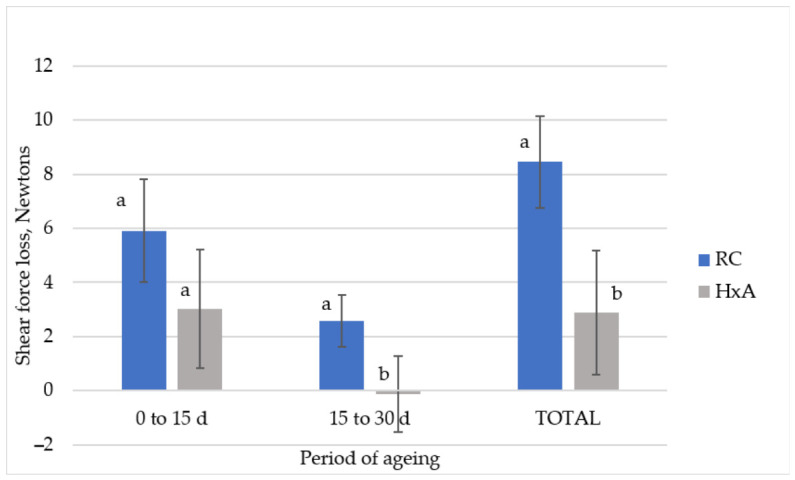
Changes in Warner–Bratzler shear force (SF) during ageing. Bars represent the loss of SF in ageing intervals (0–15, 15–30 d, and total loss) for Criollo (RC) and Hereford × Angus (H × A) beef. The data are expressed as means ± standard errors. Different letters (a, b) indicate significant differences among storage times within the same animal group (*p* < 0.05).

The RC beef showed a greater development of tenderness with ageing compared to the H × A. This is evident in the trend of a higher reduction in total SF (*p* = 0.057). While the commercial crossbreed beef lost approximately 3 N of SF primarily in the first 15 days of ageing, stabilizing until the 30th day, the RC beef lost around 8 N over the 30 days of ageing. In Figure 4 higher losses of SF across ageing represent higher tenderness in RC.

### 3.4. Colour

No significant effects of genetic group or ageing time were observed for instrumental colour coordinates (*p* > 0.05). However, a significant interaction of genetic group × ageing was detected (*p* < 0.05). Beef from commercial H × A generally exhibited higher L* values than RC beef during the early ageing stages (Table 1). However, both groups converged toward similar L* values by 30 d of ageing. RC beef displayed a temporary increase in L* at 15 d before stabilizing (Table 1). Regarding redness (a*), a significant interaction was observed (*p* < 0.05). Commercial beef (H × A) maintained stable a* values until 15 d of ageing, followed by a slight decline toward 30 d. In contrast, RC beef showed a non-significant increase in redness up to day 15 and maintained higher chromatic stability through the end of the ageing period. The crossbreed beef (H × A) exhibited a marked increase and variability in b* after 15 d of ageing, followed by a return to baseline values at 30 d, whereas RC beef displayed a more gradual increase in b* across the ageing period. Chroma (C*) followed a similar pattern in both groups, with no significant breed or interaction effects detected (*p* > 0.05).

## 4. Discussion

### 4.1. pH

Meat quality characteristics such as pH kinetics, colour stability, and textural profiles, are intrinsically linked and serve as primary determinants of consumer acceptability and industrial functionality. The *postmortem* conversion of muscle to meat is governed by complex biochemical processes, where the rate and velocity of pH decline determine the final structural and organoleptic properties of meat [25,26].

The pH of beef in the present study remained within the optimal range for high-quality beef (5.4–5.8), suggesting adequate glycogen reserves at slaughter and limited pre-slaughter stress. No significant genetic group effect was observed and RC exhibited steady pH values during ageing, indicating a comparable *postmortem* metabolic progression between both genetic groups. The maintenance of pH within this range is essential to beef because it directly influences key quality attributes, including WHC, colour stability, tenderness, flavour development, and microbial stability [13].

*Postmortem* anaerobic glycolysis in muscle facilitates the accumulation of lactic acid, changing the pH from approximately 7.2 to an ultimate pH within the range of 5.4 to 5.8. This acidification is critical, as the ultimate pH is optimal for maintaining myofibrillar integrity and enzymatic activity, thereby ensuring superior tenderness and the development of a desirable bright-red bloom [27,28]. Conversely, abnormal pH declines, whether overly rapid or insufficient, compromise protein solubility and endogenous proteolytic systems, such as the calpain–calpastatin complex. This results in suboptimal colouration and toughening. A decrease in meat quality and the potential for maintaining a high pH during cooling has been noted when the pH exceeds 5.8 after 24 h *postmortem* [29]. Consequently, a comprehensive understanding of the synergistic relationship between pH, colour, and tenderness is imperative for the delivery of high-quality products demanded by rigorous market standards [30].

Glycogen is responsible for the normal drop in muscle pH during *postmortem* metabolism. Meat with a high pH is dark in colour, dry in appearance and firm to the touch (DFD meat). On the other hand, meat with too low a pH (≤5.3) is referred to as PSE meat and is pale, exudative, and too soft. In the case of PSE meat, a decrease in pH takes place while the carcass is still warm, with a decrease below 6.0 already occurring 45 min after slaughter [31]. Neither of the two abnormal cases was observed in the present study.

### 4.2. Water-Holding Capacity

While various factors influence WHC, their effects are primarily mediated through alterations in the spatial configuration and structural integrity of muscle proteins. Water in the tissue serves as a plasticizer for muscle proteins [32].

The WHC in the present study indicated a progressive increase in water retention as ageing progressed beyond four weeks, which could be related to evaporation of moisture during the process. WHC reflects the ability of muscle tissue to retain water within its structural matrix, largely through interactions with myofibrillar and cytoskeletal proteins that bind most of the intracellular water [33]. This ability of muscle to retain intrinsic moisture is prevalent throughout the distinct *postmortem* phases of storage, handling, processing, and thermal treatment. Since lean skeletal muscle comprises approximately 75% water, WHC constitutes a critical determinant of economic value and functional quality [34]. Suboptimal WHC results in negative effects, including increased exudative drip loss within retail packaging, substantial cooking loss due to myofibrillar shrinkage, and the consequent reduction in the sensory juiciness of the final product [35].

WHC is strongly influenced by pH-dependent changes in protein structure, as reduced pH promotes partial protein denaturation and decreases the availability of water-binding sites. During the early *postmortem* period, structural contraction of myofibrils promotes the migration of immobilized water toward extracellular spaces, contributing to drip formation [12]. As ageing progresses, proteolytic degradation of cytoskeletal proteins and gradual deterioration of cell membranes further facilitate water loss that can be released from the ageing muscle [36]. The progress of WHC during extended ageing in this study is therefore consistent with previously described structural modifications occurring during meat ageing [37]. This suggests that the water losses occurring during ageing primarily affect free or immobilized water fractions rather than the structural water associated with myofibrillar structures that contribute most strongly to perceived tenderness and juiciness [38].

### 4.3. Warner–Bratzler Shear Force

SF values represent the maximum energy in Newtons to cut a sample of meat, which reflect the tenderness of the tissue—the lower the value, the more tender the beef. Tenderness is widely recognized as one of the most important determinants of beef palatability and is largely governed by *postmortem* proteolytic activity. The higher initial SF values observed in the RC group compared with the Hereford × Angus (H × A) group may reflect genetic differences in the activity of the calpain–calpastatin proteolytic system. Calpastatin, a specific inhibitor of μ- and m-calpain, has been shown to vary among breeds and can influence the rate of *postmortem* tenderization [39].

Tenderization during *postmortem* storage may depend on cattle breed. A study by Pringle et al. [40] evidenced that strip loin and top sirloin steaks from Angus and F1 Brahman × Angus steers were more tender than steaks from Brahman; however, top round steak tenderness was not different across breed type. Meat tenderness is partly determined by the calpain (CAPN1)/calpastatin (CAST) protein system. Some authors compared the genetic variability in the CAPN1 gene in Creole, Brahman and Nellore breeds in Bolivia. Creole cattle have a higher frequency of alleles associated with higher meat tenderness than the Zebu breeds. The authors recommended the use of these markers in breeding programs to improve Bolivian cattle herd meat quality either by selection within Creole breeds or crosses with Zebu cattle [41].

Although crossbred animals exhibited faster tenderization during the early ageing period, reaching a plateau around day 15, RC beef showed a more gradual but sustained and higher reduction in SF throughout the entire ageing process. This pattern suggests that proteolytic activity in Criollo muscle may remain active for a longer period during ageing, allowing continued structural weakening of myofibrillar components, resulting in higher tenderization [1]. These kinds of differences in tenderization dynamics have been previously associated with breed-dependent regulation of proteolytic enzymes and structural muscle characteristics [42].

It is important to note that even within the first three days *postmortem*, the shear force (SF) values of Raramuri Criollo (RC) beef can be classified as tender, ranging from 18 to 22 N. These values are lower than the thresholds of 29.42 N and 42.87 N reported by Miller et al. [43] and Destefanis et al. [44], respectively. Additionally, these values align with those reported by Caraveo-Suarez et al. [45] who found shear force measurements of 2.31 to 4.45 N in the L. dorsi and Triceps brachii muscles of Raramuri Criollo cattle after 15 days of storage at 4 °C.

Currently, there is no available information on the proteolytic activity, myofibrillar fragmentation index (MFI), or other biochemical processes occurring in RC beef during the ageing process. However, emerging studies on other rustic breeds have begun to provide insights. For example, research on the genetic variability of the CAPN1 gene in Bolivian Creole cattle—a breed similar to RC—has shown a higher frequency of alleles associated with more tender beef compared to Bos indicus breeds [41]. Moreover, considering the genetic origins of RC, which trace back to Iberian and Canarian regions of Europe, it is likely that they share ancestral genes with breeds like Avileña, Asturiana, or Morucha. These Spanish breeds have been studied and recognized for their ability to produce tender beef [46,47].

However, emerging evidence suggests that Criollo carcasses may not easily fit within the traditional meat grading system, as they tend to deposit limited amounts of subcutaneous fat compared to beef counterparts, but still produce desirable flavour and tenderness, though this requires more detailed study [48,49].

By day 15, SF values in both groups were already within the range commonly associated with very tender beef (WBSF < 38.25 N) [50], indicating that the ageing process effectively improved meat tenderness regardless of breed. These findings support the relevance of *postmortem* ageing as a technological strategy for enhancing the palatability of beef and demonstrate that even without extended ageing RC beef can reach tenderness levels comparable to or higher than commercial crossbred cattle [51,52]. The most important point is that tenderness parameters such as SF in meat from RC remained within ranges associated with very tender meat quality.

### 4.4. Colour

Meat colour is a critical quality attribute influencing consumer purchase decision and is primarily determined by the chemical state of myoglobin, its concentration, and the structural characteristics of the muscle [53]. During storage and ageing, oxidative processes may promote the conversion of oxymyoglobin to metmyoglobin, resulting in gradual discoloration [54].

Holman et al. [55] established that beef colour was considered acceptable when a* ≥ 14.5. The a* values observed in the present study (Table 1) are higher than that value, and in accordance with those reported by Realini et al. [56], who defined a fresh beef steak as one where the colour values are L* > 39.5, a* > 16.8, and b* > 6.3. Our results in RC show values of L* > 33.7, a* > 19.2, and b* > 9.7 at 0 d of storage, increasing during storage. Such values are among the recommended values for fresh meat. Similarly, Zhang et al. [57] defined the colour ranges of fresh beef as L* > 31.4, a* > 16.4, and b* > 6.5. Orellana et al. [58] compared the meat colour parameters of Argentine Creole cattle with European cattle, determining that the meat of Creole cattle had a darker colour than meat from European-breed cattle. Similarly, Albertí et al. [59] reported that rustic breeds such as Creole have darker meat than genetically improved breeds.

The decrease in lightness (L*) observed during ageing in beef from the commercial cross may be associated with progressive dehydration of the muscle surface and structural changes that reduce light reflectance. Similar trends have been reported in aged beef where moisture loss and protein degradation alter the optical properties of muscle tissue [60].

Redness (a*) tends to decline during storage, likely reflecting oxidative changes in myoglobin pigments and the effects of dehydration on pigment stability [61]. However, Criollo beef showed a tendency to retain higher redness values and exhibited a delayed increase in yellowness (b*), suggesting greater chromatic stability during ageing. This behaviour may be related to differences in muscle composition, antioxidant status, or feeding systems commonly associated with Criollo cattle raised under pasture-based conditions [62]. The stability of chroma (C*) throughout the ageing period further indicates that the overall intensity of meat colour was preserved, supporting the visual acceptability of both products.

A previous work on *L. dorsi* of RC steers reported average values of L* = 32, a* = 14, b* = 10, and C* = 12, which are slightly lower than those of the present study [45]. It is important to consider that variability of the L*, a*, and b* values can be attributed to factors that influence the meat colour, such as muscle, packaging, and storage time. Differences in meat colour could also be explained by structural attributes within the muscle cell [32]. The meat from RC could have promising market opportunities as Criollo meat comes from pasture beef. Meat from pasture-raised steers is more stable during retail refrigeration, possibly allowing for a longer shelf-life, and healthier for consumers compared to meat from concentrate-fed steers [63].

### 4.5. Practical Implications

These results demonstrate that beef originating from rustic breeds such as RC has quality characteristics comparable to beef from commercial crossbreeds such as Hereford × Angus, particularly regarding tenderness. In addition, dry ageing enhances key quality attributes of Criollo beef, effectively mitigating initial breed-related differences in tenderness and colour stability. The extended tenderization observed in Criollo cattle suggests that longer ageing periods may be beneficial, although further research is warranted to explore optimal ageing durations beyond 30 d. The application of ageing protocols can significantly improve the marketability of Criollo beef, elevating it to levels comparable with specialized breeds while maintaining its distinctive quality traits. Furthermore, understanding breed-specific ageing responses enables tailored *postmortem* handling strategies to maximize meat quality and consumer satisfaction.

Beyond meat quality, the study highlights the ecological and cultural relevance of Creole cattle populations maintained in arid landscapes. Their productive integration into regional livestock systems may therefore contribute to sustainable meat production strategies that balance product quality, ecosystem conservation, and rural livelihoods.

## 5. Conclusions

Our findings demonstrate that beef from Raramuri Criollo is initially tender at 48 h *postmortem*. Furthermore, dry ageing significantly improves beef tenderness without compromising key quality indicators such as colour, pH, or water-holding capacity. Consequently, dry ageing serves as a viable, low-intervention method for further enhancing the value of lean muscle from diverse bovine crossbreeds, achieving quality comparable to high-input industrial crossbreeds like Hereford × Angus. Beyond technical performance, these results refute existing stigmas regarding Raramuri Criollo beef quality, positioning this rustic breed as a competitive alternative within sustainable, local food systems.

## Figures and Tables

**Table 1:** Colour parameters (CIE L*a*b* and chroma) of muscles of Raramuri Criollo and Hereford × Angus crossbred cattle stored at different times (least square means ± standard error).

Storage Days	Breed	L*			a*			b*			C*		
0	RC	33.69 ^b^	±	2.93	19.20 ^b^	±	1.78	9.67 ^b^	±	0.86	21.51	±	1.85
	H × A	38.62 ^a^	±	2.32	20.01 ^ab^	±	1.05	10.43 ^b^	±	1.02	22.57	±	1.31
15	RC	35.53 ^a^	±	2.63	20.90 ^a^	±	1.59	10.35 ^ab^	±	2.26	23.39	±	2.03
	H × A	37.25 ^a^	±	3.04	20.06 ^ab^	±	1.05	12.74 ^a^	±	3.99	23.92	±	2.83
30	RC	35.29 ^a^	±	2.63	20.16 ^ab^	±	1.25	10.89 ^a^	±	0.76	22.93	±	1.23
	H × A	36.26 ^a^	±	2.31	19.30 ^ab^	±	2.13	10.32 ^ab^	±	1.57	21.89	±	2.59

Colour parameters of beef from Raramuri Criollo (RC) and Hereford × Angus crossbred (H × A) cattle during ageing (0, 15, and 30 d). Colour is described as coordinates: lightness (L* 100 = white, 0 = black), redness (a* ± red–green) and yellowness (b* ± yellow–blue) on the CIELab scale. Chroma (C*) was calculated from a* and b*. The data are expressed as means ± standard errors. Means with different letters within a column indicate a significant difference (*p* < 0.05) in the colour parameter among groups of beef, related to the breed × ageing time interaction effect. No statistical significance (*p* > 0.05) was detected from the main effects (breed or ageing time).

## Data Availability

The dataset is available upon request.
